# Granular Endometritis.—Menorrhagia from Fungous Endometritis.—Dysmenorrhea Due to Anteflexion

**Published:** 1894-05

**Authors:** Augustin H. Goelet

**Affiliations:** New York


### GRANULAR ENDOMETRITIS.—MENORRHAGIA FROM FUNGOUS ENDOMETRITIS.—DYSMENORRHEA DUE TO ANTEFLEXION.*

By AUGUSTIN H. GOELET, M.D.,

NEW YORK.

GENTLEMEN—The first case I show you to-day is one of granular endometritis which gives the following history:

Mrs. McC., aged twenty years, married three years, had one child eighteen months ago, but has not been pregnant since. She complains of backache, some pelvic pain, has a profuse leucorrhea and is constipated.

On examination we find the uterus occupying a position with the fundus, not completely retroverted, but lying backwards towards the sacrum, both ovaries are slightly prolapsed, especially the left, though they are not particularly sensitive to pressure. There is a slight laceration of the cervix and the lips feel irregular as if the surface was granular.. Ths uterine body is soft. Examination with the speculum shows that there is a rather abundant leucorrhea, muco-purulent in character, and there is a granular endocervitis with a similar condition of the cavity of the uterus. This may be treated in one or two ways according to modern views, either by dilatation, curettage and drainage, or by cupric electrolysis.

The first method would involve the use of an anesthetic and necessitate confinement to bed for several days. As there is no appreciable disease of the appendages to contraindicate, I shall adopt the latter method, cupric electrolysis, because the patient can be treated here in the clinic, and pursue her ordinary vocation. This method consists in using an electrode of copper within the uterus with the positive pole of the galvanic current, which results in the liberation of an astringent salt, the oxychloride of copper. In addition to its being deposited upon the mucous membrane of the uterine cavity, as the result of the cataphoric action of the current, it is driven into the tissues some distance to the diseased

---

* A clinical lecture delivered at the West Side German Clinic, New York.

glands beneath the surface. The advantage of this form of medication is readily appreciated.

If this application is made when the cervical canal is not patulous, uterine colic is extremely liable to supervene, and is due I believe to the action of the astringent upon the canal in preventing free drainage from the uterine cavity. As a precaution against the occurrence of this disagreeable feature, which so often follows intrauterine applications of astringent solutions, I will first gently dilate this canal with the steel dilator, and in order to remove the secretion from the mucous membrane I will use the negative pole for a few minutes first. You see that the secretion is thus rendered thinner and the canal is made free of secretion so that the electrode can come directly in contact with the diseased surface. Now we will change to the positive pole, turn on a current of 30m., and continue it for ten minutes. When the attempt is made to remove the electrode you see that it has become adherent, but it may be detached by a gentle rotary motion. In some instances, however, it is necessary to reverse the polarity, using the negative pole for a few minutes in order to loosen and detach the electrode. You see the surface of the metal has been acted upon, and it is covered with an apple-green salt which is readily wiped off. The electrode after being washed must be rubbed with emery cloth so it will present a bright clean surface for the next application.

Into the vagina is now inserted a loose wad of iodoform gauze to which a string has been attached. This is removed by the patient in twenty-four hours. She is ordered to report in three days, but the application is repeated only at the end of a week. Some cases are completely cured by two or three such applications, but some require more. When the condition shows improvement the strength of the current is reduced and is continued for only five minutes.

CASE 2.—The next patient I show you, Mrs. H., is twenty-six years old; she has been married eight years; has had one child and three miscarriages; the last seven months ago.

She complains of backache, severe pelvic pain, especially in the left side, radiating down the thigh and a profuse leucorrhea which is sometimes streaked with blood. Her menstruation is profuse, lasting for ten and twelve days at a time, and the interval is only two weeks. She says she was curetted after each miscarriage, but has not been well since the last. She presented herself on the last clinic day and was so extremely sensitive to digital examination that it was with difficulty that an accurate diagnosis of the condition of the appendages could be made. Bipolar faradization was administered, and she experienced complete relief from her pain for the remainder of the day, and as you have heard her say, though the pain has returned it is nothing compared with that she experienced before the application was made. This patient has a fungous endometritis, and in this case when the local condition is somewhat better, that is, when the pain has been entirely relieved, I shall dilate under an anesthetic and curette the uterine cavity. We will then have an opportunity of verifying the diagnosis, and ascertain positively if there is any disease of the appendages.

CASE 3.—The next patient, Mrs. Z., is twenty-three years old, has been married two and one-half years, and has never been pregnant. She has suffered from dysmenorrhea since puberty, and since her marriage she complains of pelvic pain. At first it was confined to the left side, but now it extends over the whole pelvis. She complains also of backache and leucorrhea. You will observe on examination that she has an anteflexion which is somewhat unusual, the flexion being confined to the body of the uterus, and the cervix is elongated.

The most satisfactory treatment of these conditions is dilatation under an anesthetic and the introduction of a straight stem or hard rubber drainage-tube which is kept in place for a week while the patient is confined to bed. Subsequent treatment might also be necessary. This is not, however, always a convenient plan of treatment for this class of patients, and for this reason we will adopt another method which will accomplish the same result, though it is slower, viz.: dilatation with the negative pole of the galvanic current. I will make this application with the patient in the lateral position because you can better observe the technique. The dorsal position is usually preferred because it saves time; but in every instance, when this method is adopted, the vagina should be irrigated with an antiseptic solution, because the electrode being introduced along the finger as a guide, without such precaution, septic material might be introduced into the cavity of the uterus. When the lateral position is employed a speculum is introduced and the cervix and os cleansed by means of a pledget of absorbent cotton saturated in the antiseptic solution held in the grasp of the dressing forceps. You will observe that considerable resistance is offered to the entrance of the electrode at the point of flexion, but by depressing the handle of the instrument towards the perineum while the current is turned on it passes the constriction after a very few moments. It is not advisable in these cases to use more than 10 or 15m. for four or five minutes, because cauterization is undesirable. The applications are repeated usually twice a week. In this case we will employ vaginal bipolar faradization afterwards to relieve the pelvic pain of which she complains, though it is not always used in these cases unless the application to the canal is followed by pain.

---

### TREATMENT OF CHRONIC HEART VALVE DISEASE.

Dr. James Tyson (*Amer. Jour. Med. Sciences*) points that relief is often obtained from the occasional use of purgatives—five to ten grains of blue mass, followed by a saline, or the continuous use of small doses—one-half to one grain thrice daily. The greater apparent effect of the infusion of digitalis is due to its use in larger dose, although it is likely to be better borne by the stomach. Strophanthus, better borne by the stomach, has been used in doses of ten minims every two hours for forty-eight hours without interruption. Caffeine in three-grain doses every three hours, in mitral regurgitation, is admirable, but is likely to produce insomnia. Sparteine in one-quarter, increased to one-half grain dose, three to five times daily, is of value if a diuretic be desired. For irregularty of heart action and palpitation, more common in mitral disease, belladonna is very useful. A belladonna plaster placed over a palpitating heart is a most efficient agent. Nitro-glycerin, one-hundredth of a grain, increased to double the quantity, three times daily, often serves to the same end.—*Med. Standard.*

